# The Microbiota Promotes Arterial Thrombosis in Low-Density Lipoprotein Receptor-Deficient Mice

**DOI:** 10.1128/mBio.02298-19

**Published:** 2019-10-22

**Authors:** Klytaimnistra Kiouptsi, Sven Jäckel, Giulia Pontarollo, Alexandra Grill, Marijke J. E. Kuijpers, Eivor Wilms, Christian Weber, Felix Sommer, Magdolna Nagy, Carlos Neideck, Yvonne Jansen, Stefanie Ascher, Henning Formes, Cornelia Karwot, Franziska Bayer, Bettina Kollar, Saravanan Subramaniam, Michael Molitor, Philip Wenzel, Philip Rosenstiel, Hristo Todorov, Susanne Gerber, Ulrich Walter, Kerstin Jurk, Johan W. M. Heemskerk, Emiel P. C. van der Vorst, Yvonne Döring, Christoph Reinhardt

**Affiliations:** a Center for Thrombosis and Hemostasis (CTH), University Medical Center Mainz, Mainz, Germany; b German Center for Cardiovascular Research (DZHK), Partner Site RheinMain, Mainz, Germany; c Department of Biochemistry, Cardiovascular Research Institute Maastricht, Maastricht University, Maastricht, The Netherlands; d Institute of Cardiovascular Prevention, Department of Medicine, Ludwig-Maximilians-University Munich, Munich, Germany; e German Center for Cardiovascular Research (DZHK), Partner Site Munich Heart Alliance, Munich, Germany; f Institute of Clinical Molecular Biology (IKMB), Kiel University, Kiel, Germany; g Center for Cardiology, Cardiology I, University Medical Center Mainz, Mainz, Germany; h Institute of Developmental Biology and Neurobiology, Johannes Gutenberg University of Mainz, Mainz, Germany; i Division of Angiology, Swiss Cardiovascular Center, Inselspital, Bern University Hospital, Bern, Switzerland; University of Wisconsin—Madison; University of Texas at Austin

**Keywords:** gut microbiota, germfree, low-density lipoprotein receptor, arterial thrombosis, atherothrombosis, carotid artery, atherosclerosis, microbiota, platelets, vascular inflammation

## Abstract

Atherosclerotic plaque development depends on chronic inflammation of the arterial wall. A dysbiotic gut microbiota can cause low-grade inflammation, and microbiota composition was linked to cardiovascular disease risk. However, the role of this environmental factor in atherothrombosis remains undefined. To analyze the impact of gut microbiota on atherothrombosis, we rederived low-density lipoprotein receptor-deficient (*Ldlr^−/−^*) mice as germfree (GF) and kept these mice for 16 weeks on an atherogenic high-fat Western diet (HFD) under GF isolator conditions and under conventionally raised specific-pathogen-free conditions (CONV-R). In spite of reduced diversity of the cecal gut microbiome, caused by atherogenic HFD, GF *Ldlr^−/−^* mice and CONV-R *Ldlr^−/−^* mice exhibited atherosclerotic lesions of comparable sizes in the common carotid artery. In contrast to HFD-fed mice, showing no difference in total cholesterol levels, CONV-R *Ldlr^−/−^* mice fed control diet (CD) had significantly reduced total plasma cholesterol, very-low-density lipoprotein (VLDL), and LDL levels compared with GF *Ldlr^−/−^* mice. Myeloid cell counts in blood as well as leukocyte adhesion to the vessel wall at the common carotid artery of GF *Ldlr^−/−^* mice on HFD were diminished compared to CONV-R *Ldlr^−/−^* controls. Plasma cytokine profiling revealed reduced levels of the proinflammatory chemokines CCL7 and CXCL1 in GF *Ldlr^−/−^* mice, whereas the T-cell-related interleukin 9 (IL-9) and IL-27 were elevated. In the atherothrombosis model of ultrasound-induced rupture of the common carotid artery plaque, thrombus area was significantly reduced in GF *Ldlr^−/−^* mice relative to CONV-R *Ldlr^−/−^* mice. *Ex vivo*, this atherothrombotic phenotype was explained by decreased adhesion-dependent platelet activation and thrombus growth of HFD-fed GF *Ldlr^−/−^* mice on type III collagen.

## Abstract (precis)

Our results demonstrate a functional role for the commensal microbiota in atherothrombosis. In a ferric chloride injury model of the carotid artery, GF C57BL/6J mice had increased occlusion times compared to colonized controls. Interestingly, in late atherosclerosis, HFD-fed GF *Ldlr^−/−^* mice had reduced plaque rupture-induced thrombus growth in the carotid artery and diminished *ex vivo* thrombus formation under arterial flow conditions.

## INTRODUCTION

Atherosclerosis, a major burden of Western lifestyle-associated diseases, is a chronic inflammatory process of the vessel wall arising at sites of disturbed blood flow. The development of atherosclerotic plaques depends on hypercholesterolemia and has been linked to low-grade inflammation-triggered by innate immune signaling (1). In addition, infection can promote lesion progression, and many studies have linked innate immunity as a possible cause of atherosclerosis (2–5). Since the gut microbiota is an established factor shaping the host’s innate immunity response, its absence and the composition of this densely colonized microbial ecosystem have been associated with atherosclerosis and arterial thrombosis (6–13).

It is known that signaling through pattern recognition receptors profoundly influences the development of atherosclerotic lesions (14). In the genetic apolipoprotein E-deficient (*Apoe^−/−^*) mouse model, lacking the Toll-like receptor (TLR) adaptor protein Myeloid differentiation primary response 88 (MyD88), a reduction in lesion size was noted (15). Likewise, Toll-like receptor 2 (*Tlr2*)-deficient hypercholesterolemic *Ldlr^−/−^* mice were shown to have only minimal lesions compared with wild-type (WT) *Ldlr^−/−^* controls (16). Furthermore, *Tlr4* deficiency in *Apoe^−/−^* mice resulted in reduced aortic atherosclerosis (17). In contrast, germfree (GF) *Apoe^−/−^* mice carrying the *lps^d^* allele, thus being unable to respond to bacterial lipopolysaccharide (LPS), did not show an altered lipid profile and had similar levels of aortic cholesterol esters and aortic root plaque size compared to their conventionally raised (CONV-R) controls (18). In spite of the wealth of genetic mouse studies, the role of the commensal gut microbiota in stimulating carotid artery atherosclerosis, the site where plaques frequently rupture and embolize, causing stroke, remains poorly understood.

The variable composition of commensal microbiota has been suggested as an environmental factor contributing to cardiometabolic disease states (6, 16), development of atherosclerotic lesions (19, 20) and arterial thrombosis (11–13, 21). We have recently demonstrated that GF mice are protected from angiotensin II-induced vascular dysfunction and angiotensin II-induced hypertension, which could ultimately contribute to the development of atherosclerosis (22). In addition, carnitine and choline nutrients that are metabolized by the commensal microbiota to yield proatherogenic trimethylamine (23), as well as exogenous bacterial (24) and endogenous TLR ligands (25) (e.g., HMGB-1) are considered to promote cardiovascular disease development. In shotgun sequencing analyses of fecal samples from patients with symptomatic atherosclerosis, *Collinsella* was found to be enriched, whereas *Roseburia* and *Eubacterium* were reduced (9). Although the commensal microbiota composition has emerged as a risk factor for cardiometabolic disease (26), it remains controversial how the gut metagenome contributes to the development of atherosclerosis (7, 18–20) and in particular how the gut microbiota influences atherothrombotic processes, i.e., the rupture of an atherosclerotic plaque at the carotid artery, with subsequent thrombus formation.

The possible contribution of commensal microbiota to Western diet-induced carotid artery atherosclerosis and plaque thrombogenicity in the *Ldlr^−/−^* mouse model is unknown. To explore whether the microbiota is involved in atherothrombosis, we comparatively analyzed the plasma lipoprotein profile, vascular inflammation, carotid artery plaque size, extent of plaque rupture-induced thrombosis, and *ex vivo* platelet adhesion to collagen under flow in GF *Ldlr^−/−^* and CONV-R *Ldlr^−/−^* mice, receiving a normal chow control diet (CD) or a high-fat Western diet (HFD) for 16 weeks, respectively. Our results demonstrate that the commensal microbiota reduced plasma cholesterol levels in CD-fed *Ldlr^−/−^* mice, but not in HFD-fed *Ldlr^−/−^* mice. The presence of the microbiota in HFD-fed *Ldlr^−/−^* mice did not affect late atherosclerotic lesion size at the carotid artery but increased myeloid blood cell counts and platelet deposition to collagen coatings, associated with increased thrombus growth in the carotid artery following ultrasound-induced plaque rupture.

## RESULTS

*Ldlr^−/−^* mice are a well-established model of familial hypercholesterolemia, characterized by 2-fold higher plasma cholesterol levels compared to WT controls on a normal CD (27). If subjected to a HFD, *Ldlr^−/−^* mice show 10-fold-higher plasma cholesterol levels, increased VLDL and LDL levels with atheroma formation in the aorta and characteristic plaque formation at the aortic root (27). Since the role of commensal microbiota on hypercholesterolemia and atherothrombosis is unresolved (19, 28), we rederived CONV-R *Ldlr^−/−^* mice as GF and kept this mouse line on a sterile gamma-irradiated HFD at GF isolator conditions for 16 weeks to compare them with CONV-R *Ldlr^−/−^* control mice (sterility control; see Fig. S1 in the supplemental material).

10.1128/mBio.02298-19.1 FIG S1 Sterility controls on the feces from GF *Ldlr^−/−^* mice. Lane 1, molecular weight DNA marker; lane 2, feces from GF *Ldlr^−/−^* mice; lane 3, positive 16S rDNA control; lane 4, negative (empty) control. Download FIG S1, PDF file, 0.03 MB. Copyright © 2019 Kiouptsi et al. 2019 Kiouptsi et al. This content is distributed under the terms of the Creative Commons Attribution 4.0 International license.

### Feeding *Ldlr*-deficient mice with atherogenic diet alters gut microbial diversity.

**FIG 1:**
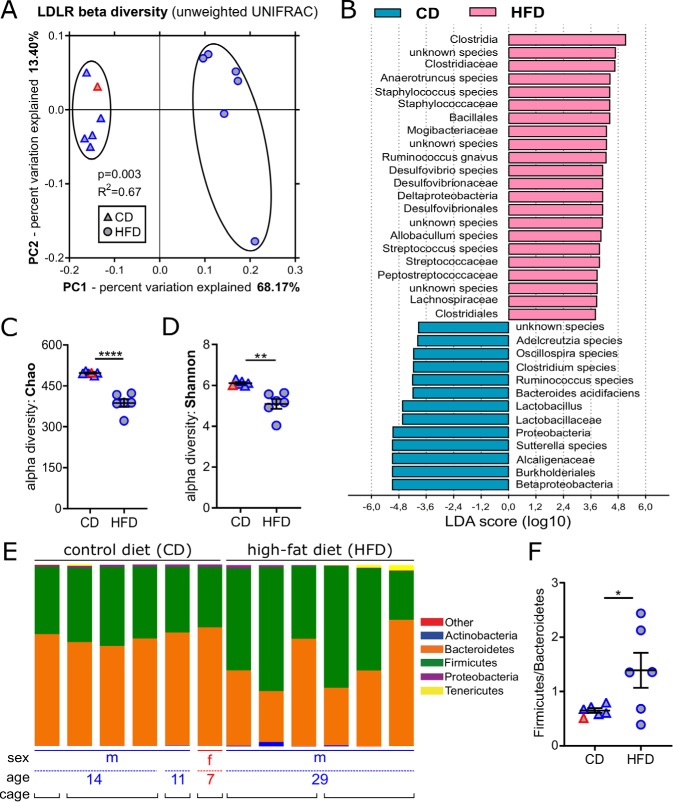
HFD feeding promotes microbiota dysbiosis in *Ldlr^−/−^* mice as revealed by cecal 16S ribosomal DNA amplicon sequencing. (A) Principal coordinate analysis reveals distinct separation in microbiota composition due to diet (*P* = 0.003 for control diet [CD] versus high-fat diet [HFD]). LDLR, LDL receptor. (B) Linear discriminant analysis (LDA score) effect size (LEfSe) highlights differentially abundant microbial taxa (CD versus HFD). (C and D) Reduced alpha diversity in HFD versus CD using Chao richness (C) and Shannon entropy (D) metrics. (E) HFD-induced changes in microbiome composition on the phylum level. (F) *Firmicutes*/*Bacteroidetes* ratio in CD and HFD samples. In panels A, C, D, and F, the sex of the mice fed CD (triangles) and HFD (circles) is color coded (females [red] and males [blue]). In panels C, D, and F, means ± standard errors of the means (SEM) (error bars) are shown for the groups. Independent samples from mice fed HFD versus CD were compared by Student *t* tests, and values that were significantly different are indicated by bars and asterisks as follows: *, *P* < 0.05; **, *P* < 0.01; ****, *P* < 0.0001.

HFD feeding was previously associated with dysbiosis that is characterized by a reduced diversity of the gut microbiota (29, 30), resulting in decreased intestinal barrier function and in the onset of a low-grade inflammatory state (20, 31). To monitor the influence of HFD feeding, we compared the cecal microbiome composition of *Ldlr^−/−^* littermates on CD with mice that at the same age were fed with an atherogenic HFD for 16 weeks. We noted vast changes in diversity between the groups (beta-diversity) (Fig. 1A). In CONV-R *Ldlr^−/−^* mice on HFD, the relative abundance of members of the taxa *Clostridiaceae*, *Staphylococcaceae*, *Bacillales*, *Streptococcaceae*, and *Clostridales* increased relative to mice fed CD, whereas *Lactobacillaceae*, *Proteobacteria*, and *Betaproteobacteria* decreased (Fig. 1B; see Table S1). Generally, the bacterial richness of the cecal microbial community was significantly reduced by HFD feeding (Fig. 1C and D). Furthermore, the ratio of *Firmicutes* to *Bacteroidetes*, the dominant bacterial divisions in the distal mouse intestine (32), was significantly increased in HFD-fed mice compared to CD-fed mice (Fig. 1E and F; Fig. S2A). Collectively, these data demonstrate that in the *Ldlr^−/−^* mouse model, HFD feeding resulted in a dysbiotic cecal microbiota.

10.1128/mBio.02298-19.2 FIG S2 Taxon summary on the genus level. Download FIG S2, PDF file, 0.2 MB. Copyright © 2019 Kiouptsi et al. 2019 Kiouptsi et al. This content is distributed under the terms of the Creative Commons Attribution 4.0 International license.

10.1128/mBio.02298-19.7 TABLE S1 Differences in microbial taxa between HFD and CD mice. Download Table S1, PDF file, 0.03 MB. Copyright © 2019 Kiouptsi et al. 2019 Kiouptsi et al. This content is distributed under the terms of the Creative Commons Attribution 4.0 International license.

### The gut microbiota modulates the plasma lipoprotein profile with CD feeding but does not alter lipoprotein composition or carotid artery atherosclerotic lesion size on HFD.

**FIG 2:**
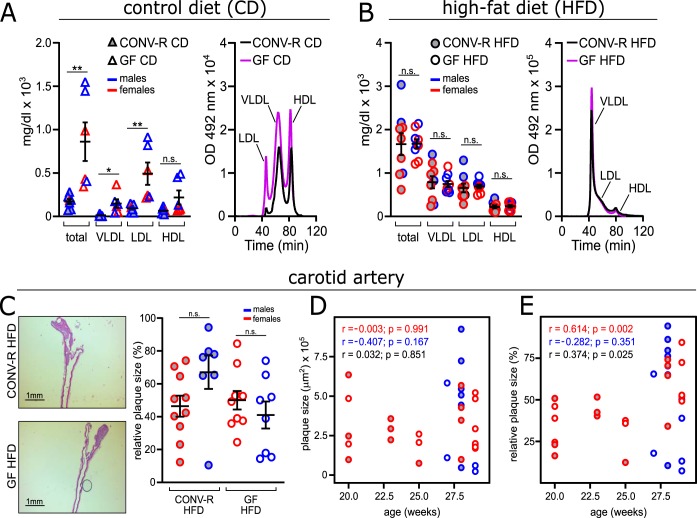
Familial hypercholesterolemia is increased in GF *Ldlr^−/−^* mice on control diet (CD), but both cholesterol levels and atherosclerotic lesion size in the carotid artery are unchanged after 16 weeks on high-fat diet (HFD) for GF *Ldlr^−/−^* and CONV-R *Ldlr^−/−^* mice. (A and B) Total cholesterol level and fractioned cholesterol levels (VLDL, LDL, and HDL) of GF *Ldlr^−/−^* and CONV-R *Ldlr^−/−^* mice on CD (8 CONV-R mice; 6 GF mice) (A) and HFD (9 mice/group) (B). For the chromatogram, representative lipoprotein profiles detected in CONV-R (black lines) and GF (purple lines) mice are shown. OD, optical density. (C, left) Representative histology images showing hematoxylin-and-eosin-stained sections of carotid artery plaques in GF and CONV-R *Ldlr^−/−^* mice on HFD. Bars, 1 mm. (Right) Relative atherosclerotic plaque size (as a percentage) of the carotid artery of GF (17 mice [9 females and 8 males]) and CONV-R (17 mice [10 females and 7 males]) *Ldlr^−/−^* mice after 16 weeks on a HFD, split by sex. Means ± SEM (error bars) are shown for the groups. Independent samples were compared by Student *t* tests. Values that were significantly different are indicated by bars and asterisks as follows: *, *P* < 0.05; **, *P* < 0.01. Values that were not significantly different (n.s.) are indicated. (D and E) Correlation between age and the absolute plaque size (in square micrometers) (D) or relative plaque size (as a percentage) (E) at the carotid artery. For all panels, data for CONV-R mice are shown as gray dots, and data for GF animals are shown as white dots. The sex of the mice is color coded as follows: females in red and males in blue.

Next, we analyzed whether the microbial colonization state of the host influences the composition of the lipoprotein profile. Similar to *Apoe^−/−^* mice (7, 28), GF *Ldlr^−/−^* mice on CD had increased total plasma cholesterol levels compared with CONV-R *Ldlr^−/−^* mice (Fig. 2A). The higher total plasma cholesterol levels in GF *Ldlr^−/−^* mice were reflected by significantly increased VLDL and LDL cholesterol, along with elevated cholesterol in high-density lipoprotein (HDL) particles. Intriguingly, this microbiota-dependent effect on the plasma cholesterol levels and the lipoprotein profile was abolished when the *Ldlr^−/−^* mice were fed atherogenic HFD for 16 weeks (Fig. 2B).

Histological analyses of fixed-frozen sections demonstrated that atherosclerotic plaque areas in the hematoxylin-and-eosin-stained carotid artery were not altered despite the absence of commensals in GF *Ldlr^−/−^* mice compared to CONV-R *Ldlr^−/−^* mice on HFD (Fig. 2C). Of note, we also did not observe altered plaque sizes or wall thickness in the common carotid artery, as determined by high-frequency ultrasound imaging (Fig. S3A to C). When stratified by sex, the relative plaque size of carotid artery plaques was not different between male and female HFD-fed *Ldlr^−/−^* mice (Fig. 2C). Furthermore, the absolute atherosclerotic lesion size in the carotid arteries did not differ between GF *Ldlr^−/−^* and CONV-R *Ldlr^−/−^* mice (Fig. 2D; Table S2).

10.1128/mBio.02298-19.3 FIG S3 High-frequency small animal ultrasound of both carotids of GF and CONV-R mice 8 weeks after HFD or CD. (A) Representative ultrasound B-images of the carotid arteries of HFD mice. (B) Measurement of the wall thickness of the anterior carotid wall from the right (RCC) and left common carotid (LCC) arteries. (C) Cumulative atherosclerotic plaque size of RCC (right) and LCC (left) in B-mode. Mean plus SEM (error bars) are shown for the groups (2 to 5 mice per group). For panels B and C, CONV-R mice are shown by gray bars, and GF animals are shown in white bars. Independent samples were tested by Student *t* test. **, *P* < 0.01. Download FIG S3, PDF file, 0.3 MB. Copyright © 2019 Kiouptsi et al. 2019 Kiouptsi et al. This content is distributed under the terms of the Creative Commons Attribution 4.0 International license.

10.1128/mBio.02298-19.8 TABLE S2 Absolute (μm^2^) and relative (%) values for atherosclerotic plaque area at the carotid artery and aortic root (zero-level) of the 40 CONV-R (gray) and GF (white) animals fed for 16 weeks with HFD. Animals are color coded as in Fig. 2C and D: males are shown in blue, while females are shown in red. Download Table S2, PDF file, 0.04 MB. Copyright © 2019 Kiouptsi et al. 2019 Kiouptsi et al. This content is distributed under the terms of the Creative Commons Attribution 4.0 International license.

To investigate whether age significantly impacts atherosclerotic lesion size of the *Ldlr^−/−^* mice fed HFD for 16 weeks, we calculated the Pearson correlation between age and plaque size in the carotid artery. Age did not significantly correlate with absolute plaque size in the carotid artery but affected the relative values (Fig. 2D and E). In order to investigate whether relative plaque size in the carotid artery differs between GF *Ldlr^−/−^* and CONV-R *Ldlr^−/−^* mice after adjusting for age differences, we performed an analysis of covariance (ANCOVA) with group assignment and sex as factors and age as covariate. The effect of age was statistically significant (*F*_(1, 32)_ = 7.002, *P* = 0.0125). However, there was no significant difference between male and female animals (*F*_(1, 32)_ = 0.48, *P* = 0.493) and between CONV-R and GF mice (*F*_(1, 32)_ = 1.41, *P* = 0.244) even after adjusting for the effect of age. In conclusion, the age of *Ldlr^−/−^* mice influenced relative plaque size, but the GF housing condition had no significant influence on late atherosclerotic lesion size in the carotid artery after 16 weeks of HFD feeding.

### Germfree *Ldlr*-deficient mice on HFD show reduced vascular inflammation.

**FIG 3:**
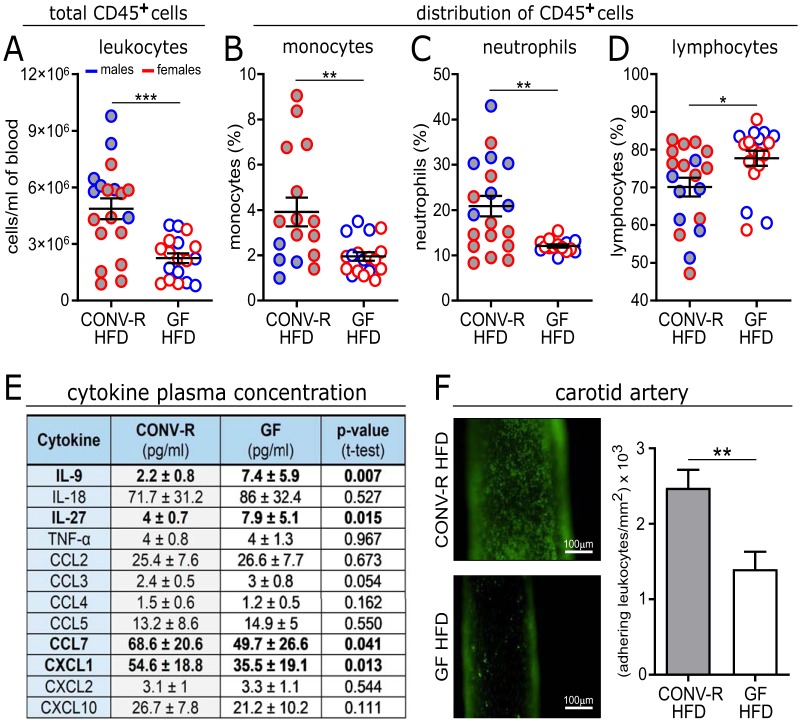
GF *Ldlr^−/−^* mice show reduced vascular inflammation after 16 weeks on HFD. (A to D) Blood cell counts of total leukocytes (18 or 19 mice/group) (A) and percentage of CD45^+^ cells: monocytes (16 to 18 mice/group) (B), neutrophils (15 to 19 mice/group) (C), and lymphocytes (18 or 19 mice/group) (D) from GF and CONV-R *Ldlr^−/−^* mice on HFD for 16 weeks, analyzed by flow cytometry. Means ± SEM (error bars) are shown for the groups. (E) Multiplex cytokine ELISA quantification of mouse plasma samples (6 to 16 mice/group). (F) Intravital epifluorescence video microscopy of endothelial adherent and rolling leukocytes (green) in the common carotid artery of GF and CONV-R *Ldlr^−/−^* mice on HFD for 16 weeks (11 to 16 mice/group). Nucleated cells were visualized with acridine orange. Means ± SEM are shown for the groups. Independent samples were compared by Student *t* tests. Statistical significance: *, *P* < 0.05; **, *P* < 0.01; ***, *P* < 0.001. For all panels, data for CONV-R mice are shown in gray, and data for GF animals are shown in white. For panels A to D, the sex of the mice is color coded as follows: females in red and males in blue.

To assess the vascular inflammatory phenotype of the generated gnotobiotic mouse model in late atherosclerosis, we next analyzed blood cell counts of GF *Ldlr^−/−^* mice on HFD relative to CONV-R controls. Feeding on a HFD for 16 weeks increased white blood cell counts in CONV-R *Ldlr^−/−^* mice relative to GF *Ldlr^−/−^* mice about 2-fold (Fig. 3A). The distribution of CD45^+^ cells revealed that the microbiota-dependent increase in leukocyte counts in CONV-R *Ldlr^−/−^* mice was most prominent in the myeloid lineage, as shown for monocytes and neutrophils (2-fold increase) (Fig. 3B and C). In addition, lymphocyte numbers were slightly increased in HFD-fed GF Ldlr^−/−^ mice (Fig. 3D). In contrast, platelet counts were unchanged (not shown). Consistent with the increased myeloid cell numbers, an altered plasma cytokine profile was detected, featuring increased levels of monocyte-derived proinflammatory chemokines CCL7 (chemokine [C-C motif] ligand 7) and neutrophil-derived CXCL1 (chemokine [C-X-C motif] ligand 1) in CONV-R *Ldlr^−/−^* mice, whereas the T-cell-related interleukins IL-9 and IL-27 were reduced in CONV-R *Ldlr^−/−^* mice relative to GF *Ldlr^−/−^* mice (Fig. 3E). In line with decreased plasma CCL7 and CXCL1 levels and reduced leukocyte counts quantified in the HFD-fed GF *Ldlr^−/−^* mice compared to HFD-fed CONV-R *Ldlr^−/−^* mice, intravital imaging of the carotid artery wall demonstrated that the number of adhering leukocytes to the arterial endothelium of HFD-fed GF *Ldlr^−/−^* mice was significantly reduced relative to CONV-R *Ldlr^−/−^* controls (Fig. 3F). Thus, our data indicate that the microbiota enhances vascular inflammation at the carotid artery of HFD-fed *Ldlr^−/−^* mice.

### The microbiota promotes carotid artery thrombosis.

**FIG 4:**
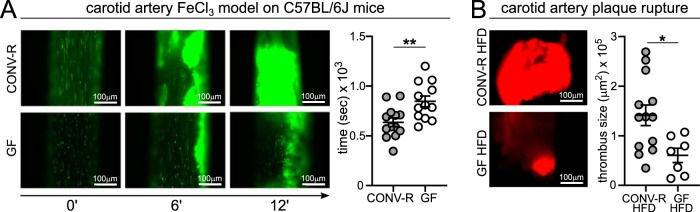
Reduced FeCl_3_-induced carotid artery occlusion in GF C57BL/6J mice and reduced plaque rupture-induced thrombogenicity in GF *Ldlr^−/−^* mice. (A) Intravital epifluorescence video microscopy of thrombus formation (DCF-stained platelets [green]) at 0, 6, and 12 min in the 10% FeCl_3_-injured common carotid artery of GF (12 mice) and CONV-R (13 mice) C57BL/6J wild-type mice (representative images) with analysis of occlusion times. (B) Intravital epifluorescence video microscopy of thrombus formation (Rhodamin B-stained platelets [red]) in the common carotid artery of GF (7 mice) and CONV-R (13 mice) *Ldlr^−/−^* mice on HFD after plaque rupture induced by 5 min of ultrasound. Means ± SEM are shown for the groups. Independent samples were analyzed by Student *t* tests. Statistical significance was indicated by asterisks as follows: *, *P* < 0.05; **, *P* < 0.01. For all panels, CONV-R mice are shown in gray, and GF animals are shown as white dots.

The gut microbiota is a factor that promotes ligation injury-induced thrombosis in the carotid artery of C57BL/6J mice on a normal chow diet (12). Interestingly, in the ferric chloride (FeCl_3_) injury model of the common carotid artery, GF C57BL/6J mice kept on a CD also showed reduced thrombus growth compared to CONV-R C57BL/6J controls, as shown by significantly prolonged occlusion times (846.7 ± 54.5 s in GF mice relative to 635.1 ± 43.8 s in CONV-R mice) (Fig. 4A). Likewise, also ex-GF mice that were colonized for 14 days with a cecal gut microbiota from a CONV-R donor mouse (conventionally derived) presented significantly reduced occlusion times (639.6 ± 64.5 s) compared to GF mice (846.7 ± 54.5 s) (data not shown). Our experimental data on GF wild-type C57BL/6J mice indicate that the microbiota promotes arterial thrombus growth, but whether the microbiota also supports thrombus growth in atherothrombosis, where subendothelial matrix components from the ruptured atherosclerotic plaque come into contact with platelets, is currently unknown.

To assess whether the influence of the gut microbiota on platelet reactivity (11, 21) impacts atherosclerotic plaque thrombogenicity *in vivo*, we applied an established ultrasound-induced plaque rupture model of the carotid artery plaque, resulting in local collagen exposure (33, 34). Importantly, we found that following ultrasound-induced plaque rupture, thrombus growth was reduced by approximately 60% in GF mice compared to CONV-R *Ldlr^−/−^* mice on a HFD (Fig. 4B). Collectively, our intravital imaging results demonstrate that the presence of gut microbiota enhances ferric chloride-induced carotid artery thrombosis in C57BL/6J mice on a CD and plaque rupture-induced thrombus growth at the carotid artery plaque of late atherosclerotic *Ldlr^−/−^* mice.

### The microbiota promotes adhesion-induced platelet activation in *Ldlr*-deficient mice receiving HFD.

**FIG 5:**
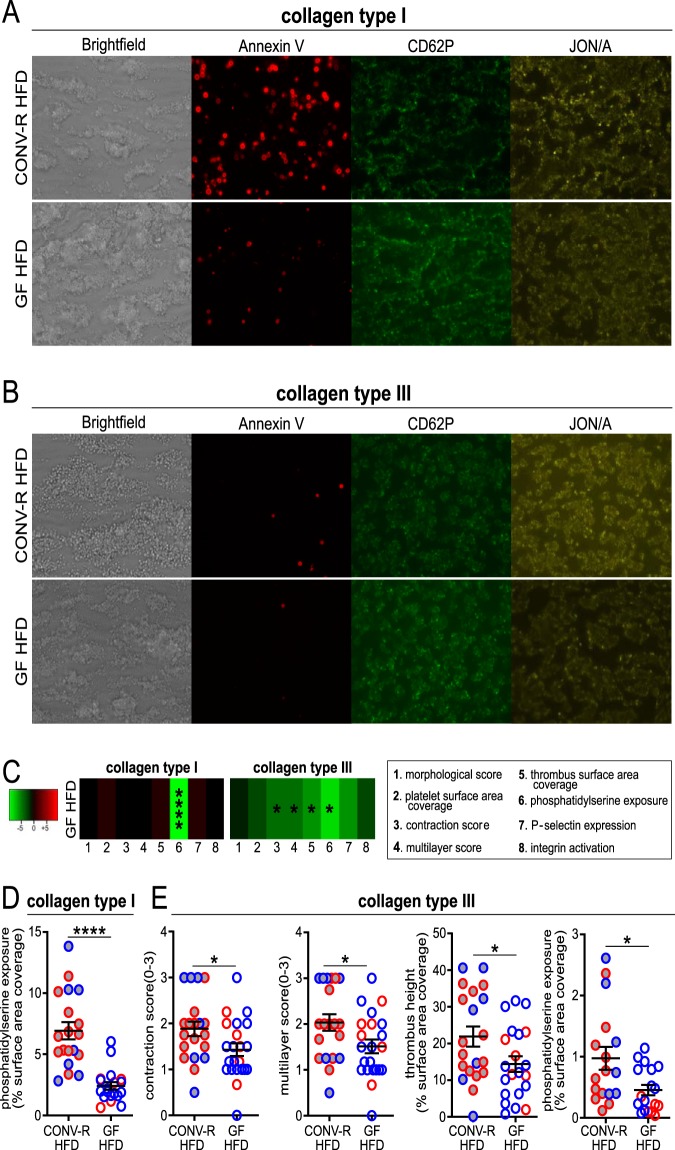
Standardized whole-blood flow chamber analysis for platelet deposition on collagen type I (A) and collagen type III (B). End-stage representative images of whole-blood platelet deposits after 3.5 min on collagen type I (A), and collagen type III (B). (C) Subtraction heatmap of HFD-fed GF *Ldlr^−/−^* mice (12 mice/group) compared to CONV-R *Ldlr^−/−^* mice (11 mice/group) (not shown). The degree of reduction relative to CONV-R *Ldlr^−/−^* mice is indicated in green (see scale panel). The analyzed parameters are as follows: 1, morphological score; 2, platelet surface area coverage; 3, thrombus contraction score; 4, multilayer score; 5, thrombus surface area coverage; 6, phosphatidylserine exposure; 7, P-selectin expression; 8, integrin α_IIb_β_3_ (GPIIbIIIa) activation. (D and E) Descriptive statistics of HFD-fed GF and CONV-R *Ldlr^−/−^* mice on collagen type I (D) and collagen type III (E) are shown only for significant results. Means ± SEM are shown for the groups. Independent samples were tested by Student *t* tests. Statistical significance is indicated as follows: *, *P* < 0.05; ****, *P* < 0.0001. For all panels, data for CONV-R mice are shown as gray dots, and data for GF animals are shown as white dots. The sex of the mice is color coded as follows: females in red and males in blue.

**FIG 6:**
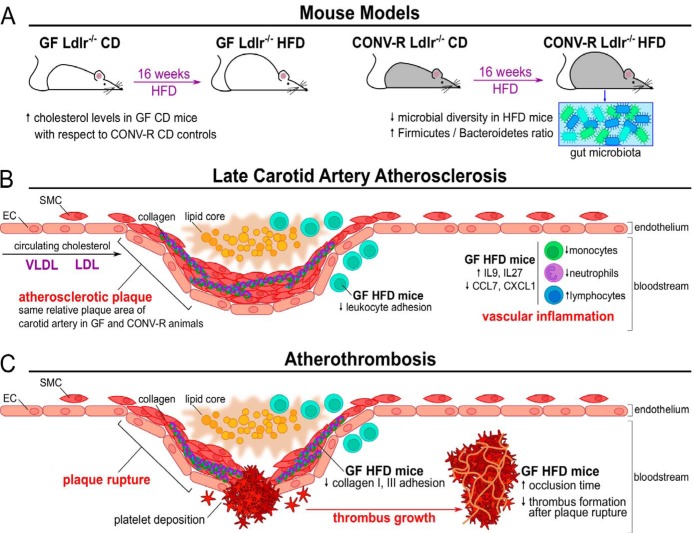
Effects of gut microbiota on late carotid artery atherosclerosis and atherothrombosis. (A) For this study, either GF or CONV-R *Ldlr^−/−^* mice on a conventional diet (CD) were fed for 16 weeks with a high-fat diet (HFD), thus resulting in the onset and progression of atherosclerosis. For CONV-R *Ldlr^−/−^* mice, the HFD yielded a reduced diversity of the commensal microbiota, with an increase in the *Firmicutes*/*Bacteroidetes* ratio. Interestingly, when fed with a CD, GF *Ldlr^−/−^* mice presented higher cholesterol levels with respect to CONV-R counterparts. (B) After cholesterol accumulation, resulting from deposition of VLDL and LDL lipoproteins, an atherosclerotic plaque presents a subendothelial lipid core with infiltrating leukocytes characterized by the accumulation of smooth muscle cells (SMC) from the tunica intima and tunica adventitia of the blood vessels. Although the relative plaque sizes at the carotid artery were unchanged between the two groups, GF animals showed altered vascular inflammatory parameters and immune cell populations. EC, endothelial cells. (C) During atherothrombosis, the plaque rupture yields to platelet deposition to the exposed subendothelial collagen, and subsequently to a bloodstream-circulating thrombus growing on the platelet plug. In this context, GF *Ldlr^−/−^* mice fed with a HFD presented lower yields of plaque rupture, collagen adhesion, and thrombus growth. Arterial occlusion time was increased in CD-fed GF C57BL/6J mice compared to CONV-R controls.

As collagen is the major platelet-activating constituent of atherosclerotic plaques (35, 36), we wanted to pinpoint whether platelet adhesion of HFD-fed GF *Ldlr^−/−^* mice to collagen is reduced compared with HFD-fed CONV-R *Ldlr^−/−^* mice. For this purpose, we used an *ex vivo* microspot-based flow chamber model, allowing multiparameter assessment of thrombus formation upon whole-blood perfusion over collagen type I and type III coatings, resulting in precise characterization of the thrombotic process and the relevant platelet activation markers (37) (Fig. 5; Fig. S5). Indeed, supporting the reduced thrombus growth *in vivo*, in this model of arterial flow-dependent thrombus formation in anticoagulated whole blood, HFD-fed GF mice showed a reduced phosphatidylserine (PS)-positive surface area coverage on type I and type III collagen compared to the blood of HFD-fed CONV-R *Ldlr^−/−^* mice (Fig. 5A to D). On type III collagen, a reduced thrombus contraction score, multilayer score, and thrombus height were observed (Fig. 5C and E). Other parameters of platelet adherence to type I and type III collagen, such as platelet deposition, morphological score, and P-selectin surface area coverage were unchanged in this model (Fig. 5A and B; Fig. S5). Of note, activation of the GPIIb/IIIa (integrin α_IIb_β_3_) was decreased on type III collagen in platelets from GF *Ldlr^−/−^* mice compared to CONV-R *Ldlr^−/−^* mice when fed CD (Fig. S6P), which is consistent with the defect of GF mice in integrin-dependent static adhesion of platelets to laminin in CD-fed mice (12). The presence of gut microbiota facilitates adhesion-induced platelet activation and *ex vivo* thrombus formation primarily to the type III collagen in the HFD-fed *Ldlr^−/−^* mice (Fig. 6).

10.1128/mBio.02298-19.4 FIG S4 (A) Standardized whole-blood flow chamber analysis for platelet deposition on collagen type I and collagen type III. (A) Subtraction heatmap of control diet (CD)-fed GF *Ldlr^−/−^* mice (14 mice/group) compared to CONV-R *Ldlr^−/−^* mice (12 mice/group). The degree of reduction relative to CONV-R *Ldlr^−/−^* mice is indicated in green. The numbers below the panels indicate the following parameters: 1, morphological score; 2, platelet surface area coverage; 3, thrombus contraction score; 4, multilayer score; 5, thrombus surface area coverage; 6, phosphatidylserine exposure; 7, P-selectin expression; 8, integrin α_IIb_β_3_ (GPIIbIIIa) activation. Independent samples were tested by Student *t* tests. *, *P* < 0.05. (B and C) End-stage representative images of whole-blood platelet deposits after 3.5 min on collagen type I (B) and collagen type III (C). Download FIG S4, PDF file, 0.7 MB. Copyright © 2019 Kiouptsi et al. 2019 Kiouptsi et al. This content is distributed under the terms of the Creative Commons Attribution 4.0 International license.

10.1128/mBio.02298-19.5 FIG S5 Descriptive statistics on the standardized whole-blood flow chamber analysis for platelet deposition of HFD-fed GF *Ldlr^−/−^* and CONV-R *Ldlr^−/−^* mice on collagen type I and collagen type III. Means ± SEM are shown for groups. For all the panels, CONV-R animals are shown as black dots, while GF animals are shown as white dots. Download FIG S5, PDF file, 0.1 MB. Copyright © 2019 Kiouptsi et al. 2019 Kiouptsi et al. This content is distributed under the terms of the Creative Commons Attribution 4.0 International license.

10.1128/mBio.02298-19.6 FIG S6 Descriptive statistics on the standardized whole-blood flow chamber analysis for platelet deposition of CD-fed (control diet fed) GF *Ldlr^−/−^* and CONV-R *Ldlr^−/−^* mice on collagen type I and collagen type III. Means ± SEM are shown. Independent samples were tested by Student *t* tests. **, *P* < 0.01. For all the panels, CONV-R animals are shown as black triangles, while GF animals are shown as white triangles. Download FIG S6, PDF file, 0.1 MB. Copyright © 2019 Kiouptsi et al. 2019 Kiouptsi et al. This content is distributed under the terms of the Creative Commons Attribution 4.0 International license.

## DISCUSSION

Even if hypercholesterolemia is the major factor in the onset of atherosclerotic plaques, the gut microbiota, an actuating variable of the innate immune response, could affect the progression of atherosclerosis and subsequent atherothrombosis. Feeding *Ldlr^−/−^* mice atherogenic HFD for 16 weeks resulted in a significantly reduced diversity of the cecal gut microbiota compared to feeding mice CD. In accordance with previous reports, this resulted in a decrease of the *Bacteroidetes* phylum and an increase in *Firmicutes* in the HFD group compared with littermate controls kept on a CD (38, 39). Importantly, HFD feeding was linked to changes in the gut microbiota associated with impaired intestinal barrier function and increased gut microbiota-dependent metabolic endotoxemia (31).

Total plasma cholesterol, VLDL, LDL, and HDL cholesterol levels were unchanged in CONV-R *Ldlr^−/−^* mice compared to GF *Ldlr^−/−^* mice after 16 weeks of HFD. Likewise, GF and CONV-R HFD-fed *Ldlr^−/−^* mice, receiving the same HFD for 2 weeks, did not show differences in total cholesterol levels (40). However, with basal CD feeding conditions, the absence of commensal microbiota evoked a striking increase in plasma cholesterol levels in the GF *Ldlr^−/−^* mice. Our data therefore indicate a spill-over of cholesterol resulting from the HFD feeding, which may mask the bacterial effect. Our results on increased plasma cholesterol levels in GF *Ldlr^−/−^* mice relative to CONV-R *Ldlr^−/−^* mice fed CD are in accordance with a number of reports on increased free cholesterol levels, as well as an increase in the concentration of cholesterol esters in GF *Apoe^−/−^* C57BL/6J mice (7, 28, 41). Thereby, our results demonstrate that the cholesterol-lowering effect of the gut microbiota is not specific to the *Apoe^−/−^* hyperlipidemia mouse model. This could be due to enhanced microbiota-dependent cholesterol synthesis and reduced bile acid synthesis (41). Indeed, it is most likely that the microbiota-dependent conversion of primary to secondary bile acids plays a central role under steady-state conditions (42). The involvement of the gut microbiome in the regulation of cholesterol levels is further substantiated by a clinical study demonstrating that several bacterial taxa correlated with plasma cholesterol levels in atherosclerotic patients (8). Based on our findings, it will be interesting to dissect which specific members of the microbiota promote cholesterol excretion and how their relative abundance can be modulated through targeted dietary or probiotic intervention strategies. Coherent with previous studies on GF *Apoe^−/−^* mice kept on hypercholesterolemic diet (7, 18, 28), we found the absolute size of late atherosclerotic plaques in the carotid artery unchanged between HFD-fed GF *Ldlr^−/−^* mice and HFD-fed CONV-R *Ldlr^−/−^* mice. Therefore, it will be interesting to analyze in future work whether the microbiota modulates early atherosclerosis. Moreover, the commensal microbiota is composed of both pathogenic and protective bacteria, which could also explain why the absence of microbiota did not yield in significant changes in the atherosclerotic lesion size.

Although the absolute size of the carotid artery lesions was not affected, we observed signs of elevated low-grade inflammation in CONV-R *Ldlr^−/−^* mice compared to GF *Ldlr^−/−^* mice fed HFD. This was indicated by increased white blood cell counts, namely, neutrophils and monocytes, along with elevated plasma CCL7 and CXCL1 levels. In accordance, both CCL7 and its receptor CCR2, as well as CXCL1 have been implicated in monocyte mobilization from the bone marrow in steady state and under conditions of hyperlipidemia, respectively (43, 44). Nevertheless, this did not result in increased lesional macrophage content in plaques of mice receiving 16 weeks of a HFD. Furthermore, increased levels of T-cell-related IL-9 and IL-27 were detected in the plasma of GF *Ldlr^−/−^* mice. In the *Ldlr^−/−^* mouse model, IL-27 had an inhibitory role on atherogenesis as it inhibited bone marrow-derived cell activation in the arterial walls (45). In contrast, IL-9 was functionally linked to aggravated atherosclerosis in *Apoe^−/−^* mice by the induction of vascular cell adhesion molecule 1 expression (46). In line with elevated myeloid blood cell counts, enhanced vascular inflammation was apparent by intravital imaging of the luminal side of carotid artery plaques prior to ultrasound-induced rupture, with substantially increased leukocyte accumulation. Interestingly, in the *Apoe^−/−^* mouse atherosclerosis model, this vascular inflammatory phenotype in the atheroma-prone regions of the carotid artery was demonstrated to depend on the endothelial expression of the Notch effector recombination signal binding for immunoglobulin kappa J region (RBPJ) (47), but the microbiota-dependent impact on endothelial pathways promoting leukocyte adhesion in atherosclerosis needs further investigation.

To investigate the impact of the gut microbiota in atherothrombosis, we exploited ultrasound-induced carotid artery plaque rupture combined with intravital imaging of thrombus growth (33). Our data revealed a marked reduction in thrombus size in the HFD-fed GF *Ldlr^−/−^* mice compared with their HFD-fed CONV-R *Ldlr^−/−^* counterparts, pointing to a stimulatory effect of the gut microbiota on experimental atherothrombosis. This finding expands on previous work from our laboratory, demonstrating that the presence of commensals promotes arterial thrombus growth in the ligation-injured carotid artery (12). The role of the gut microbiota in arterial thrombosis is further corroborated by our data with the carotid artery ferric chloride injury model, showing a significantly prolonged time course of thrombus growth in GF C57BL/6J mice relative to CONV-R wild-type controls. To mimic physiological arterial flow conditions, we analyzed thrombus formation on collagen type I and type III, the major platelet-activating matrix constituents of carotid artery plaques (35), with a sensitive standardized whole-blood flow chamber system to detect differences in thrombus growth and adhesion-dependent platelet activation (37). Similar to our *in vivo* observations, we found reduced thrombus height, contraction score, and multilayer score of thrombi formed on type III collagen in the anticoagulated whole blood from HFD-fed GF *Ldlr^−/−^* mice compared to HFD-fed CONV-R *Ldlr^−/−^* mice. Exposure of phosphatidylserine was likewise decreased on collagen type I and type III coatings, which is known to be mediated via signaling of the collagen receptor glycoprotein VI (GPVI) (36, 48), a crucial receptor for platelet adhesion and aggregate formation at the arterial injury site (49–51). Since we uncovered a microbiota-dependent increase in adhesion-induced platelet activation, it will be most interesting to study platelet GPVI signaling related to phosphatidylserine exposure in the GF hyperlipidemic *Ldlr^−/−^* mouse model.

In conclusion, our data demonstrate that the microbiota does not modulate late absolute atherosclerotic lesion size in the carotid artery, thus supporting recent reports using the *Apoe^−/−^* mouse model (7, 18, 28). Here we confirmed in the *Ldlr^−/−^* mouse hypercholesterolemia model that the gut microbiota reduces plasma cholesterol levels with CD feeding, but not under HFD-induced hypercholesterolemia in the *Ldlr^−/−^* mouse model (7, 28). Our results demonstrate that, despite not affecting the absolute lesion area in the carotid artery, the commensal microbiota augments low-grade inflammation in the vessel wall. Our results suggest that a diminished adhesion-dependent platelet activation on type I and type III collagen causes reduced plaque rupture atherothrombosis in the carotid artery of HFD-fed GF *Ldlr^−/−^* mice. Future experiments should provide mechanistic insights on how the gut microbiota interferes with platelet-collagen interaction, the pivotal pathomechanism in arterial thrombosis.

## MATERIALS AND METHODS

### Animals.

B6.129S7Ldlrtm1Her/J mice (*Ldlr^−/−^* mice) (52) were purchased from The Jackson Laboratory (Bar Harbor, ME, USA). *Ldlr^−/−^* mice were rederived as germfree (GF) by aseptic hysterectomy and maintained as a GF mouse colony in sterile flexible film mouse isolator systems. The germfree status of mice was tested weekly by PCR for detection of 16S rDNA and by bacterial culture. All experimental animals were 9- to 29-week-old male or female mice housed in the Translational Animal Research Center (TARC) of the University Medical Center Mainz under specific-pathogen-free (SPF, CONV-R) or GF conditions in EU type II cages with two to five cage companions with standard autoclaved lab diet and water *ad libitum*, 22°C ± 2°C room temperature, and a 12-h light/dark cycle. All groups of mice were sex and age matched, and all mice were free of clinical symptoms. All procedures performed on mice were approved by the local committee on legislation on protection of animals (Landesuntersuchungsamt Rheinland-Pfalz, Koblenz, Germany; 23177-07/G12-1-100; 23177-07/G13-1-072, 23177-07/G16-1-013, and 23 177-07/A 18-1-005 OEW).

### Treatment of mice.

*Ldlr^−/−^* mice were fed 16 weeks with an adjusted calories diet (42% kcal from fat, 17.3% protein, 48.5% carbohydrates, 21.2% [wt/wt] fat, and 0.2% cholesterol; contains 34% [wt/wt] sucrose) that had been vacuum packaged, irradiated, and microbial analyzed (catalog no. TD.88137; Envigo, Venray, the Netherlands). Control mice were kept on an autoclaved control diet (catalog no. 5021-3; PMI Nutrition International).

### Microbiota analysis.

MiSeq 16S amplicon V4-V5 sequence data were analyzed using MacQIIME v1.9.1 (http://www.wernerlab.org/software/macqiime) (53) as described previously (54, 55). Briefly, all sequencing reads were trimmed, keeping only nucleotides with a Phred quality score of at least 20, then paired-end assembled, and mapped onto the different samples using the barcode information. Sequences were assigned to operational taxonomic units (OTUs) using uclust and the greengenes reference database (gg_13_8 release) with 97% identity. Representative OTUs were picked and taxonomy assigned using uclust and the greengenes database. Quality filtering was performed by removing chimeric sequences using ChimeraSlayer and by removing singletons and sequences that failed to align with PyNAST. The reference phylogenetic tree was constructed using FastTree 2. All samples within a single analysis were normalized by rarefaction to the minimum shared read count to account for differential sequencing depth among samples (95,000 sequences per sample). Relative abundance was calculated by dividing the number of reads for an OTU by the total number of sequences in the sample. Alpha diversity measures were computed, and unweighted Unifrac beta diversity was calculated and visualized by principal coordinate analysis. Significance of differences in abundances of various taxonomic units between control (CD) and HFD groups was calculated using *t* test, and *P* values were adjusted for multiple testing using false-discovery rate (FDR) correction (q-value). Linear discriminant analysis (LDA) effect size (LEfSe) was performed using the online tool available at http://huttenhower.sph.harvard.edu/galaxy/.

### Lipoprotein profile analysis.

Plasma samples were subjected to fast-performance liquid chromatography (gel filtration on Superose 6 column; GE Healthcare). Different lipoprotein fractions were separated and evaluated based on flowthrough time. Cholesterol levels were quantified using an enzymatic assay (Cobas, Roche) according to the manufacturer’s protocol.

### Analysis of carotid artery atherosclerotic lesion using histology.

Lesion development in the left common carotid artery was quantified by hematoxylin-and-eosin (H&E) staining of 4-μm longitudinal cryosections.

### Hematologic analysis.

EDTA anticoagulated mouse whole blood was collected from anesthetized mice (5 mg/kg midazolam [5 mg of midazolam per kg of body weight], 0.5 mg/kg medetomidine, 0.05 mg/kg fentanyl), by intracardial puncture. Platelet and total and differential white blood cell counts were determined using an automatic cell counter (ADVIA; Siemens, Erlangen, Germany).

### Flow cytometry.

Whole blood was collected in an EDTA-buffered tube, subjected to red blood cell lysis, and centrifuged for 5 min at 300 × *g*, and cell pellets were subsequently stained with different antibody cocktails for analysis by flow cytometry. Flow cytometry was conducted with a FACS Canto II, using FACSDiva software (BD Biosciences). Cell populations were discriminated by the following antibody cocktail: anti-CD45 (clone 30-F11; eBioscience), anti-CD115 (clone AFS98; eBioscience), anti-Gr1 (clone RB6-8C5; Biolegend), anti-CD11b (clone M1/70; eBioscience), anti-B220 (clone RA3-6B2; eBioscience), and anti-CD3 (clone 145-2C11; eBioscience). Cell populations and marker expression were gated using the FlowJo analysis program (Treestar): leukocytes (CD45^+^), neutrophils (CD45^+^ CD115-Gr1^high^), monocytes (CD45^+^ CD11b^+^ CD115^+^), and lymphocytes (CD45^+^ CD3^+^ and CD45^+^ B220^+^).

### Cytokine analysis.

Cytokine levels in mouse plasma samples were measured using the ProcartaPlex Multiplex Immunoassay technology from Affymetrix (Affymetrix, eBioscience, ProcartaPlex Mouse Cytokine & Chemokine Panel 1, 26-plex), according to the manufacturer’s protocol.

### Preparation of platelets and leukocytes for intravital epifluorescence microscopy.

Mice were anesthetized by intraperitoneal injection of a solution of midazolame (5 mg/kg body weight), medetomidine (0.5 mg/kg body weight), and fentanyl (0.05 mg/kg body weight). Citrated whole blood was collected by intracardial puncture. Murine platelets were isolated and labeled with Rhodamin B (20 μg/ml). The labeled platelet suspension was adjusted to a final concentration of 150 × 10^3^ platelets/μl, and 250 μl of suspension was injected via a jugular vein catheter. Thrombus formation of murine platelets was assessed by high-speed epifluorescence microscopy 10 min after plaque rupture. To characterize leukocyte adhesion and rolling *in vivo*, leukocytes stained with acridine orange (50 μg/μl; 100 μl per mouse; Sigma-Aldrich) were imaged.

### Mouse common carotid artery thrombosis models.

Plaque rupture and measurement of acute thrombus formation were performed as described earlier (33). *Ldlr^−/−^* mice were fed high-fat diet with 42% from fat for 16 weeks. After the mice were anesthetized by intraperitoneal injection of a solution of midazolame (5 mg/kg; Ratiopharm GmbH, Ulm, Germany), medetomidine (0.5 mg/kg; Pfizer Deutschland GmbH, Berlin, Germany), and fentanyl (0.05 mg/kg; Janssen-Cilag GmbH, Neuss, Germany), a polyethylene catheter (0.28-mm inner diameter [ID], 0.61-mm outer diameter [OD]; Smiths Medical Deutschland GmbH, Grasbrunn, Germany) was implanted into the right jugular vein, and the right carotid artery was dissected free from surrounding tissue. The animal was injected intravenously with Rhodamin B-labeled platelets obtained from a donor mouse with the same genetic background, hygiene status, and feeding procedure. A subset of mice were also injected with acridine orange for leukocyte staining.

The ferric chloride injury model was performed by placing a 1-mm^2^ filter paper that was soaked with 10% (wt/wt) FeCl_3_ solution for 3 min laterally to the common carotid artery (55). Prior to the ferric chloride injury, the anesthetized mouse was infused with 5- (and 6-) carboxy-2′,7′-dichlorofluorescein diacetate (DCF)-stained platelets (green), as previously described (12). Directly after 3 min, the filter paper and any residuals were flushed away with isotonic sodium chloride solution that was warmed to 37°C. Then, the common carotid artery of the mouse was placed under the objective, and the resulting thrombus formation was recorded until the artery was completely occluded by using a high-speed wide-field Olympus BX51WI fluorescence microscope with a long-distance condenser and a 10× (numerical aperture [NA] of 0.3) water immersion objective with a monochromator (MT 20E; Olympus Deutschland GmbH, Hamburg, Germany) and a charge-coupled-device camera (ORCA-R2; Hamamatsu Photonics, Japan). The time to occlusion of the FeCl_3_-injured common carotid artery was determined. If the carotid artery did not occlude within 45 min, the experiment was terminated. Videos were recorded before the application of the filter paper, directly after removing the filter paper, and then at 3-min intervals.

### Intravital high-speed video epifluorescence microscopy.

Using intravital fluorescence microscopy, a plaque was selected, and a rupture was induced by ultrasound application using a VibraCell VCX130 processor (Sonics, Newtown, CT, USA) (33, 34). Measurements were performed with a high-speed wide-field Olympus BX51WI fluorescence microscope using a long-distance condenser and a 10× (NA of 0.3) water immersion objective with a monochromator (MT 20E; Olympus Deutschland GmbH, Hamburg, Germany) and a charge-coupled-device camera (ORCA-R2; Hamamatsu Photonics, Japan). For image acquisition and analysis, the Realtime Imaging System eXcellence RT (Olympus Deutschland GmbH, Hamburg, Germany) software was used. Thrombus formation was recorded as soon as possible by capturing fluorescent images for at least 10 min. For image analysis, a threshold level was set, and thrombus area was measured.

### Whole-blood thrombus formation and platelet adhesion under flow.

Experiments were performed as described previously (56) with minor modifications. Glass coverslips (24 by 60 mm) were coated with two microspots (0.5 μl/spot) in the direction of the flow: (i) collagen type III (100 μg/ml; Octapharma, Berlin, Germany) and (ii) collagen type I (100 μg/ml; Nycomed Pharma, Munich, Germany). The coated coverslips were blocked with modified Tyrode’s HEPES buffer (pH 7.45) (TH buffer) (5 mM HEPES, 136 mM NaCl, 2.7 mM KCl, 2 mM MgCl_2_, 0.42 mM NaH_2_PO_4_) containing 1% bovine serum albumin (BSA) and mounted in parallel plate flow chambers.

Blood samples were collected by retro-orbital puncture of mice under isoflurane anesthesia into 40 μM PPACK, 5 U/ml heparin, and 50 U/ml fragmin (final concentrations) and perfused for 3.5 min, at a wall shear rate of 1,000 s^−1^, over the microspot coatings. The platelets were stained by 2 min after perfusion with Tyrode’s HEPES buffer (pH 7.45) (5 mM HEPES, 136 NaCl, 2.7 mM KCl, 2 mM MgCl_2_, 0.42 NaH_2_PO_4_, 2 mM CaCl_2_, 1 mg/ml glucose, 1 U/ml heparin, and 1 mg/ml BSA), supplemented with fluorescein isothiocyanate (FITC)-labeled rat anti-mouse CD62P monoclonal antibody (MAb) (1:40) (Emfret Analytics, Würzburg, Germany), phycoerythrin (PE)-labeled rat anti-mouse JON/A MAb (1:20) (Emfret Analytics), and Alexa Fluor 647 (AF647)-labeled annexin A5 (1:200) (Invitrogen Life Technologies, Carlsbad, CA, USA). Representative end-stage bright-field microscopic images were taken from each microspot during staining. After 2 min of stasis, remaining labels were washed away by perfusion with label-free Tyrode’s HEPES buffer, after which three representative end-stage fluorescence images were collected per microspot.

Microscopic images (1,360 by 1,024 pixels, 142 by 107 μm, 8-bit) were recorded with an EVOS fluorescence microscope (Life Technologies, Carlsbad, CA, USA), equipped with green fluorescent protein (GFP), red fluorescent protein (RFP), and Cy5 light-emitting diode (LED) cubes, and an Olympus 60× oil immersion objective, basically as described previously (37). Recorded images were analyzed using semiautomated scripts written in Fiji software (Laboratory for Optical and Computational Instrumentation at the University of Wisconsin—Madison, USA). This resulted in percentages of surface area coverage (%SAC) of deposited platelets/thrombus (from bright-field images) and of %SAC of platelet/thrombus fluorescence per fluorescent label. In addition, bright-field images were analyzed for a morphological score (scale of 0 to 5), thrombus contraction score (scale of 0 to 3), and thrombus height (multilayer, scale of 0 to 3) in comparison to reference images, to provide an indication of the overall size and height of platelet aggregates on the microspots (37). Finally, the %SAC of multilayered thrombi was analyzed by manual coloring in Fiji.

For each flow run, parameter values from individual bright-field and fluorescence images were averaged to obtain one value per parameter per microspot. These values were linearly normalized to a scale from 0 to 10 per individual parameter. Gene effect heatmaps were obtained by subtracting the normalized average values per parameter of GF mice minus CONV-R mice. Differences compared to CONV-R mice were considered statistically significant with *P* < 0.05 (*t* test, two-sided, equal variance) after correction for multiple comparisons, where required. Subtraction heatmaps were produced by using the R package version 3.2.5 (www.r-project.org).

### Statistical analysis.

Data are presented as means ± standard errors of the means (SEM). Statistical calculations were performed with GraphPad Prism 5 (GraphPad Software Inc., San Diego, CA, USA) using the independent samples Student’s *t* test to compare two groups and analysis of variance (ANOVA) with Tukey *post hoc* test for more than two groups.

### Availability of data and materials.

Sequence files and metadata for all samples used in this study have been deposited in the ENA database (https://www.ebi.ac.uk/ena) under the accession numbers ERS2865886 to ERS2865897. The applied commands and the LDA effect size are provided as supplemental material (Text S1). Other data sets used and/or analyzed during the current study are available from the corresponding author upon request.

10.1128/mBio.02298-19.9 TEXT S1 Commands. Download TEXT S1, DOCX file, 0.1 MB. Copyright © 2019 Kiouptsi et al. 2019 Kiouptsi et al. This content is distributed under the terms of the Creative Commons Attribution 4.0 International license.
