# Supplementary figures and images for: The Microbiota Promotes Arterial Thrombosis in Low-Density Lipoprotein Receptor-Deficient Mice
Source: mBio. 2019 Oct 22;10(5):e02298-19. doi: 10.1128/mBio.02298-19 (PMC6805995; doi:10.1128/mBio.02298-19)

# Suppl. Figure 1

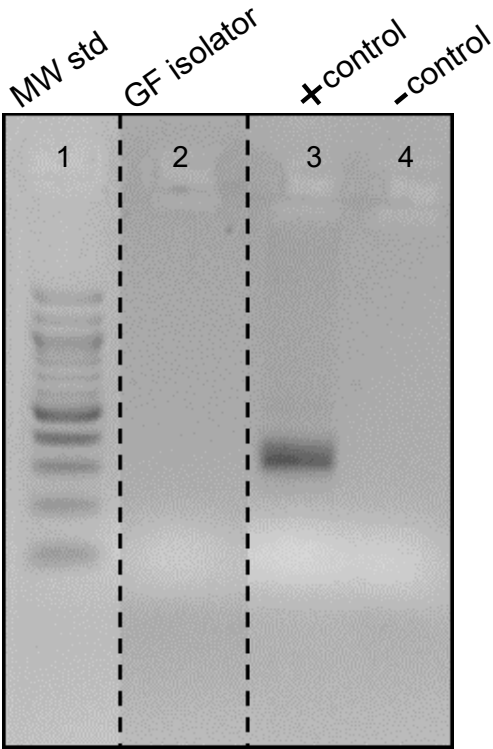

Supplement: FIG S1 [file mBio.02298-19-sf001.pdf]

# Suppl. Figure 2

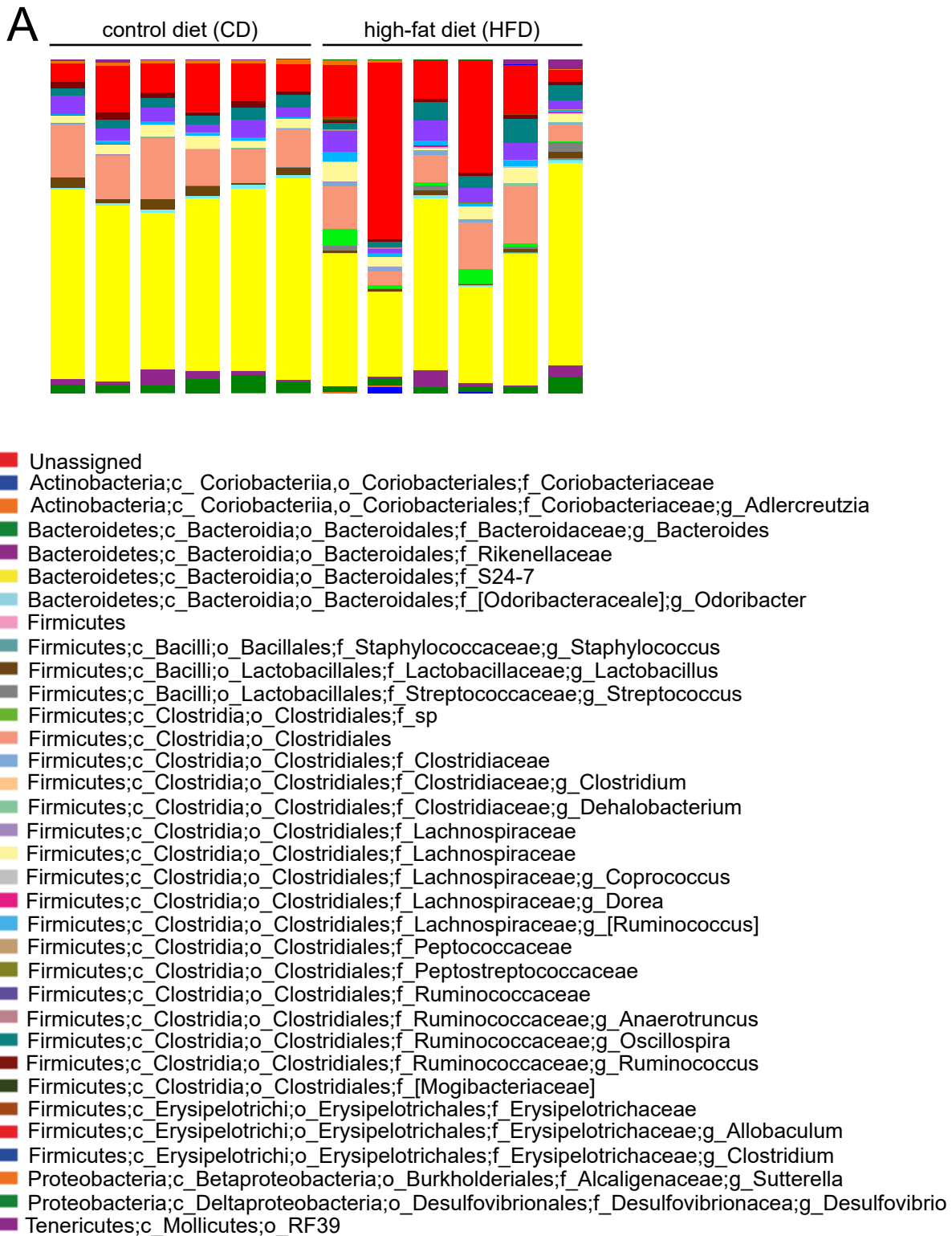

Supplement: FIG S2 [file mBio.02298-19-sf002.pdf]

# Suppl. Figure 3

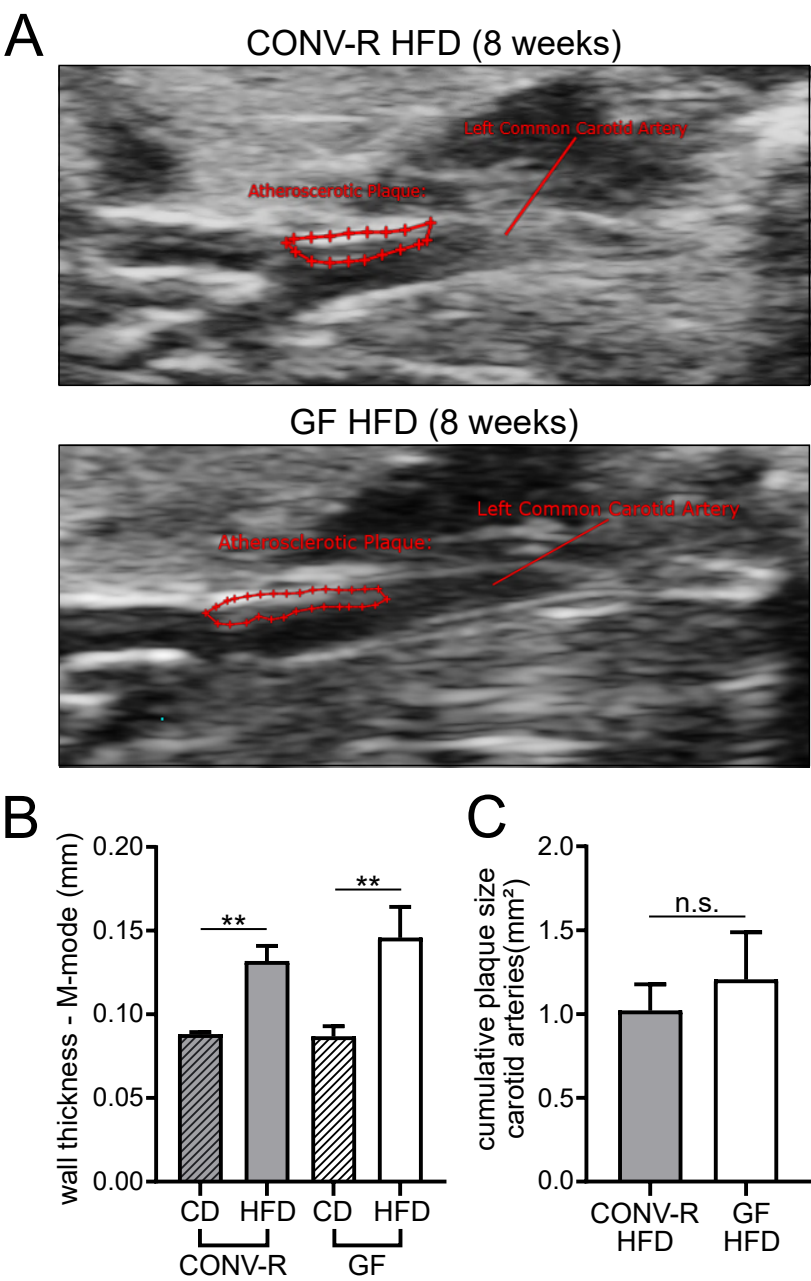

Supplement: FIG S3 [file mBio.02298-19-sf003.pdf]

# Suppl. Figure 4

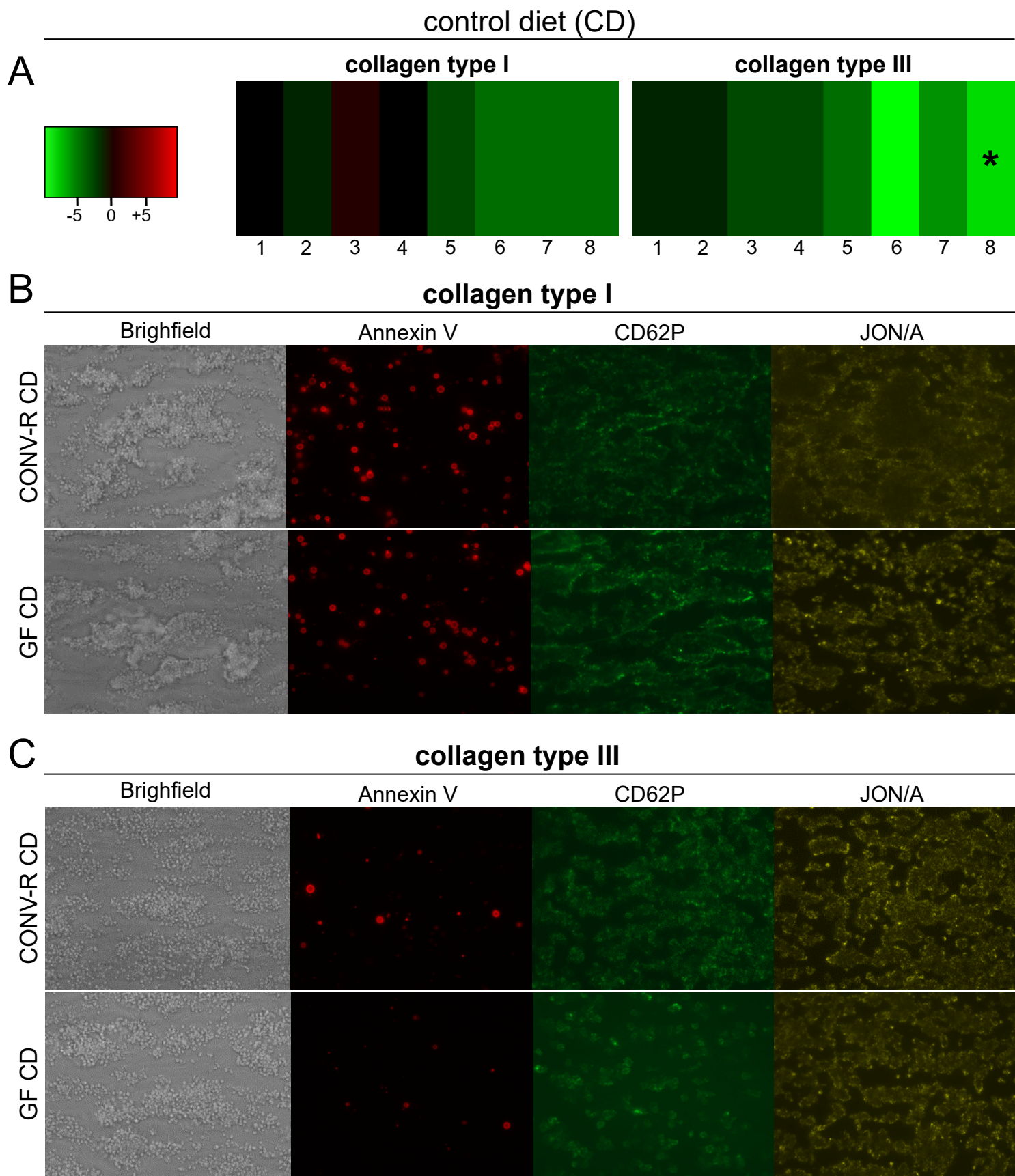

Supplement: FIG S4 [file mBio.02298-19-sf004.pdf]

# Suppl. Figure 5

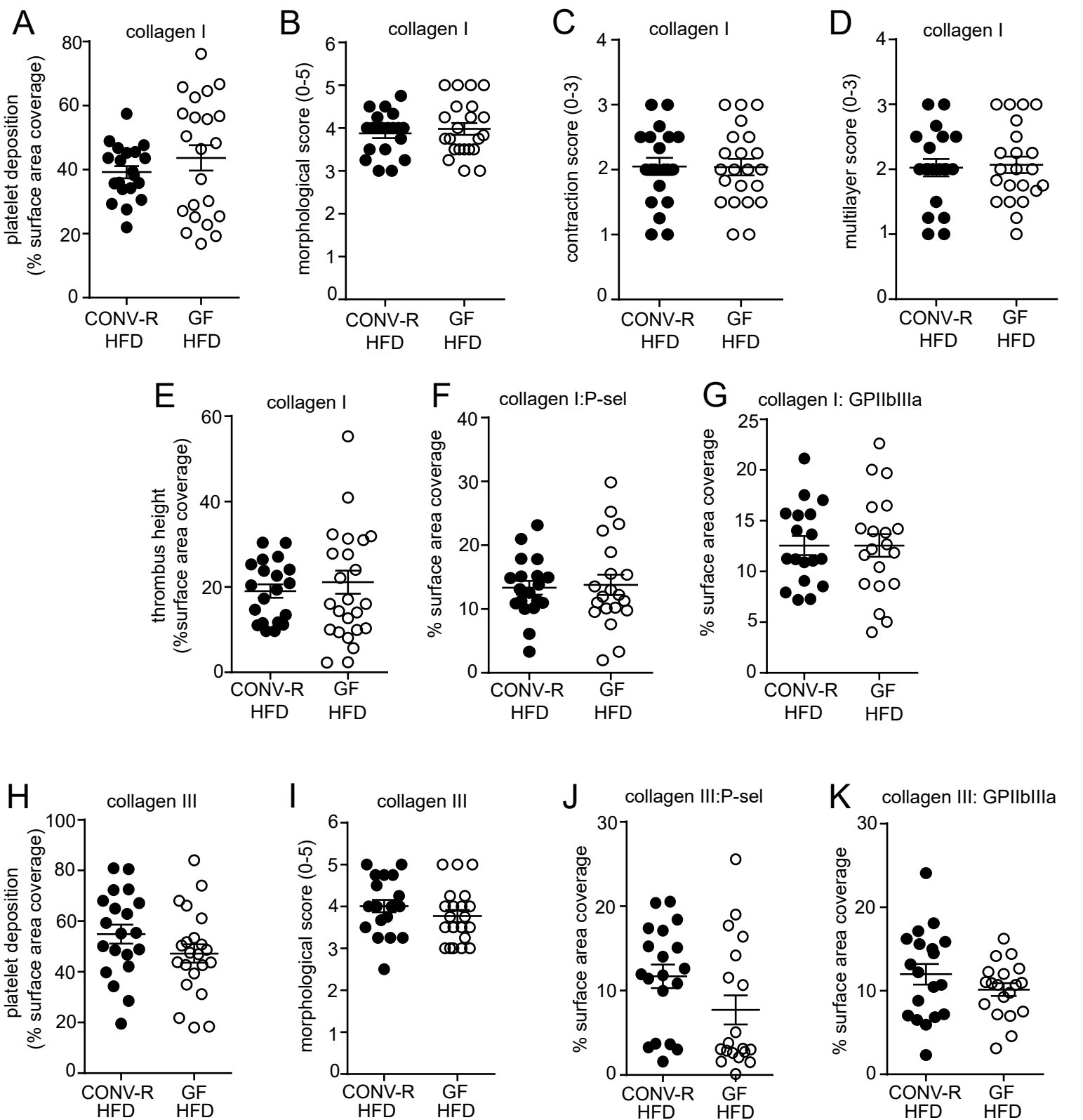

Supplement: FIG S5 [file mBio.02298-19-sf005.pdf]

# Suppl. Figure 6

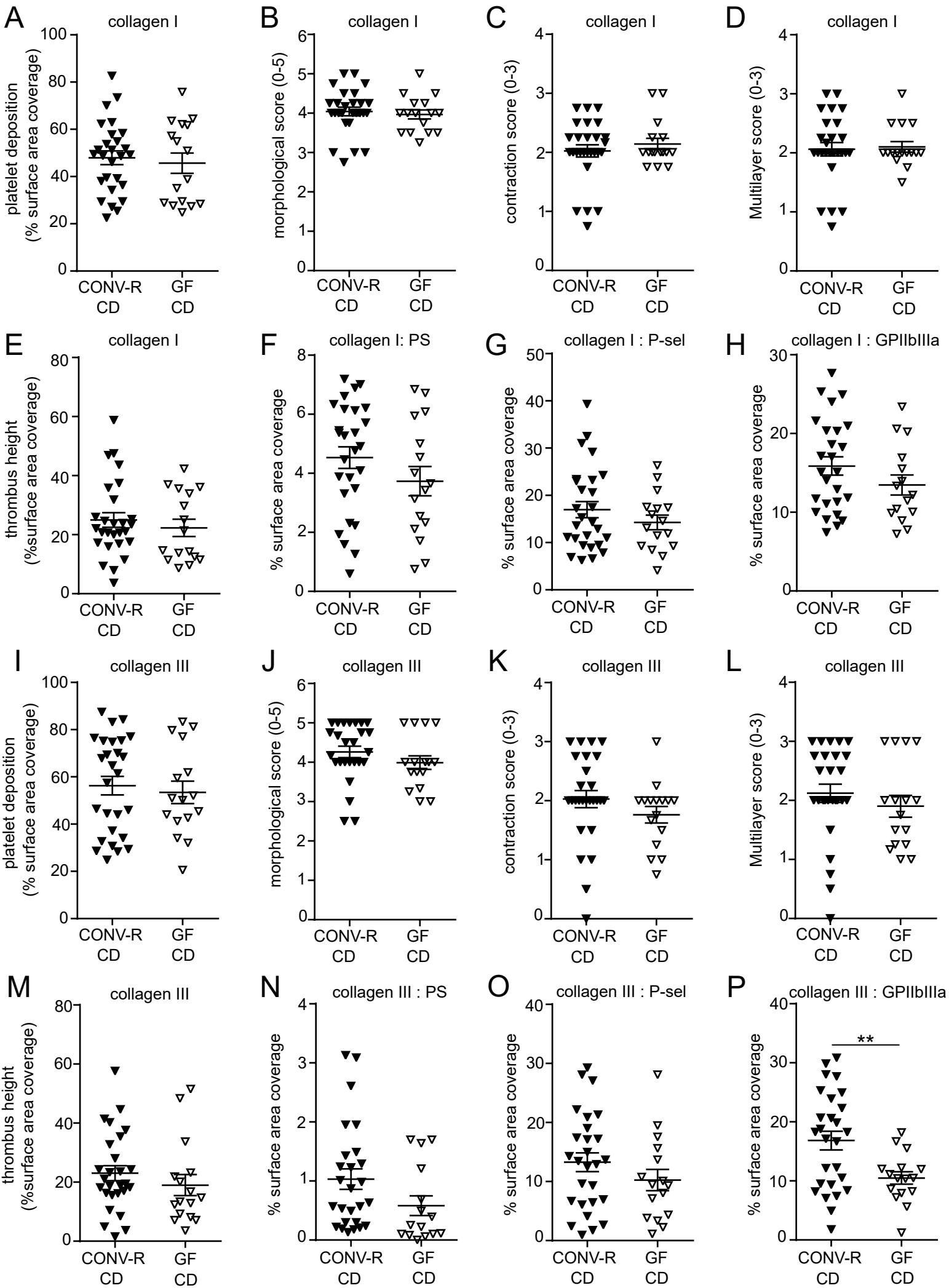

Supplement: FIG S6 [file mBio.02298-19-sf006.pdf]
