# Supplementary material for: The Microbiota Promotes Arterial Thrombosis in Low-Density Lipoprotein Receptor-Deficient Mice
Source: mBio. 2019 Oct 22;10(5):e02298-19. doi: 10.1128/mBio.02298-19 (PMC6805995; doi:10.1128/mBio.02298-19)
Supplement: TABLE S1 [file mBio.02298-19-st001.pdf]

Taxon	Group	LDA score (log10)	p
Bacteria.Firmicutes.**Clostridia**	**HFD**	5.12685705	0.00394775
Bacteria.Firmicutes.Clostridia.Clostridiales.Clostridiaceae.**unknown_spec_**	**HFD**	4.6825023	0.002093
Bacteria.Firmicutes.Clostridia.Clostridiales.**Clostridiaceae**	**HFD**	4.66351586	0.00394775
Bacteria.Firmicutes.Clostridia.Clostridiales.Ruminococcaceae.**Anaerotruncus_spec_**	**HFD**	4.44262181	0.0131951
Bacteria.Firmicutes.Bacilli.Bacillales.Staphylococcaceae.**Staphylococcus_spec_**	**HFD**	4.43452227	0.00280167
Bacteria.Firmicutes.Bacilli.Bacillales.**Staphylococcaceae**	**HFD**	4.43451631	0.00280167
Bacteria.Firmicutes.Bacilli.**Bacillales**	**HFD**	4.43444953	0.00280167
Bacteria.Firmicutes.Clostridia.Clostridiales._Mogibacteriaceae_**.unknown_spec_**	**HFD**	4.29851849	0.00394775
Bacteria.Firmicutes.Clostridia.Clostridiales.**_Mogibacteriaceae**	**HFD**	4.29851849	0.00394775
Bacteria.Firmicutes.Clostridia.Clostridiales.Lachnospiraceae.**_Ruminococcus_gnavus**	**HFD**	4.27620646	0.03704073
Bacteria.Proteobacteria.Deltaproteobacteria.**Desulfovibrionales**	**HFD**	4.12798083	0.00394775
Bacteria.Proteobacteria.**Deltaproteobacteria**	**HFD**	4.12798083	0.00394775
Bacteria.Proteobacteria.Deltaproteobacteria.Desulfovibrionales.**Desulfovibrionaceae**	**HFD**	4.12798083	0.00394775
Bacteria.Proteobacteria.Deltaproteobacteria.Desulfovibrionales.Desulfovibrionaceae.**Desulfovibrio_spec_**	**HFD**	4.12798083	0.00394775
Bacteria.Firmicutes.Clostridia.Clostridiales.Lachnospiraceae.**unknown_spec_**	**HFD**	4.11639352	0.00739713
Bacteria.Firmicutes.Erysipelotrichi.Erysipelotrichales.Erysipelotrichaceae.**Allobaculum_spec_**	**HFD**	4.05499724	0.01556764
Bacteria.Firmicutes.Bacilli.Lactobacillales.**Streptococcaceae**	**HFD**	3.98462481	0.00388491
Bacteria.Firmicutes.Bacilli.Lactobacillales.Streptococcaceae.**Streptococcus_spec_**	**HFD**	3.98462481	0.00388491
Bacteria.Firmicutes.Clostridia.Clostridiales.**Peptostreptococcaceae**	**HFD**	3.88191846	0.00739713
Bacteria.Firmicutes.Clostridia.Clostridiales.Peptostreptococcaceae.**unknown_spec_**	**HFD**	3.88191841	0.00739713
Bacteria.Firmicutes.Clostridia.Clostridiales.**Lachnospiraceae**	**HFD**	3.86242255	0.02776836
Bacteria.Firmicutes.Clostridia.**Clostridiales**	**HFD**	3.81337633	0.00739713
Bacteria.Firmicutes.Erysipelotrichi.Erysipelotrichales.Erysipelotrichaceae.**unknown_spec_**	**CD**	-3.9437408	0.00739713
Bacteria.Actinobacteria.Coriobacteriia.Coriobacteriales.Coriobacteriaceae.**Adlercreutzia_spec_**	**CD**	-3.9779805	0.002093
Bacteria.Firmicutes.Clostridia.Clostridiales.Ruminococcaceae.**Oscillospira_spec_**	**CD**	-4.1630114	0.00334562

Bacteria.Firmicutes.Clostridia.Clostridiales.Clostridiaceae.**Clostridium_spec_**	**CD**	-4.1691042	0.002093
Bacteria.Firmicutes.Clostridia.Clostridiales.Ruminococcaceae.**Ruminococcus_spec_**	**CD**	-4.1826426	0.002093
Bacteria.Bacteroidetes.Bacteroidia.Bacteroidales.Bacteroidaceae.**Bacteroides_acidifaciens**	**CD**	-4.2055195	0.00394775
Bacteria.Firmicutes.Bacilli.Lactobacillales.Lactobacillaceae.**Lactobacillus**	**CD**	-4.6445094	0.00476268
Bacteria.Firmicutes.Bacilli.Lactobacillales.**Lactobacillaceae**	**CD**	-4.6485962	0.01630917
Bacteria.**Proteobacteria**	**CD**	-5.0615875	0.03737299
Bacteria.Proteobacteria.**Betaproteobacteria**	**CD**	-5.0869826	0.01630917
Bacteria.Proteobacteria.Betaproteobacteria.**Burkholderiales**	**CD**	-5.0869826	0.01630917
Bacteria.Proteobacteria.Betaproteobacteria.Burkholderiales.**Alcaligenaceae**	**CD**	-5.0869826	0.01630917
Bacteria.Proteobacteria.Betaproteobacteria.Burkholderiales.Alcaligenaceae.Sutterella_spec_	**CD**	-5.0869826	0.01630917

Supplementary Table 1. Differences in microbial taxa between HFD and CD mice, as shown in **Figure 1B**.
