# Supplementary material for: The Microbiota Promotes Arterial Thrombosis in Low-Density Lipoprotein Receptor-Deficient Mice
Source: mBio. 2019 Oct 22;10(5):e02298-19. doi: 10.1128/mBio.02298-19 (PMC6805995; doi:10.1128/mBio.02298-19)
Supplement: TABLE S2 [file mBio.02298-19-st002.pdf]

Mouse	Diet	CONV-R vs GF	Sex	Area carotid plaque (μm²)	% carotid plaque
1	**HFD**	CONV-R	m	923861.1	86.25
2	**HFD**	CONV-R	m	545789.3	76.22
3	**HFD**	CONV-R	m	723086.15	94.33
4	**HFD**	CONV-R	m	441455	79.72
5	**HFD**	CONV-R	m	506881.2	65.01
6	**HFD**	CONV-R	m	47184.4	10.52
7	**HFD**	CONV-R	m	443334.85	70.37
8	**HFD**	CONV-R	f	98754.6	34.13
9	**HFD**	CONV-R	f	567471.1	70.58
10	**HFD**	CONV-R	f	346924.3	64.62
11	**HFD**	CONV-R	f	427869.7	74.16
12	**HFD**	CONV-R	f	-	-
13	**HFD**	CONV-R	f	223247.75	40.31
14	**HFD**	CONV-R	f	293033.2	42.43
15	**HFD**	CONV-R	f	356851.8	51.65
16	**HFD**	GF	m	-	-
17	**HFD**	GF	m	60287.2	13.13
18	**HFD**	GF	m	22826.9	7.3
19	**HFD**	GF	m	197080.05	38.92
20	**HFD**	GF	m	182789.4	50.11
21	**HFD**	GF	f	293556.6	51.97
22	**HFD**	GF	f	183557.65	52.3
23	**HFD**	GF	f	170469.45	52.54
24	**HFD**	GF	f	521304.45	84.5
25	**HFD**	GF	f	199373.2	52.79
26	**HFD**	GF	f	177137.4	49.23
27	**HFD**	GF	f	483968.65	72.44
28	**HFD**	GF	f	-	-
29	**HFD**	GF	f	206849.05	37.53
30	**HFD**	GF	f	259470.2	35.84
31	**HFD**	GF	f	-	-
32	**HFD**	GF	m	582817.6	65.48
33	**HFD**	GF	m	110232.9	17.86
34	**HFD**	GF	f	196880	24.46
35	**HFD**	GF	f	247345.1	38.96
36	**HFD**	GF	f	485075.4	46.04
37	**HFD**	CONV-R	f	75126.8	12.35
38	**HFD**	CONV-R	f	246914.1	23.1
39	**HFD**	CONV-R	f	634793.8	50.87
40	**HFD**	CONV-R	f	98119.3	16.33

Supplementary Table 2. Absolute and relative values for atherosclerotic plaque area at the carotid artery of the 40 CONV-R (in grey) and GF (in white) animals fed with HFD. Animals are color-coded as in **Figure 2 (C-E)**: males are listed in blue, while females in red.
