# Supplementary material for: The Microbiota Promotes Arterial Thrombosis in Low-Density Lipoprotein Receptor-Deficient Mice
Source: mBio. 2019 Oct 22;10(5):e02298-19. doi: 10.1128/mBio.02298-19 (PMC6805995; doi:10.1128/mBio.02298-19)
Supplement: TEXT S1 [file mBio.02298-19-s0001.docx]

**#Join paired end reads**

join_paired_ends.py -f *_L001_R1_001.fastq -r *_L001_R2_001.fastq

**#OTU picking**

pick_open_reference_otus.py -i ~/CAU/Sequencing/Mainz/merged_fasta/seqs.fna -m uclust -r /macqiime/anaconda/lib/python2.7/site-packages/qiime_default_reference/gg_13_8_otus/rep_set/97_otus.fasta -s 0.1 -o ~/CAU/Sequencing/Mainz/pick_otu_uclust_gg97

#filter OTU table for 0.05% of reads per sample

filter_otus_from_otu_table.py -i Otu_table.biom --min_count_fraction 0.00005 -o Otu_table_mincount005.biom

biom summarize-table -i Otu_table_mincount005.biom -o Otu_table_mincount005.biom_summary_table.txt

biom convert -i Otu_table_mincount005.biom -o Otu_table_mincount005.biom.txt --to-tsv --header-key taxonomy

#Num samples: 12

#Num observations: 573

#Total count: 1913294

#Table density (fraction of non-zero values): 0.765

#

#Counts/sample summary:

# Min: 95033.0

# Max: 191259.0

# Median: 169379.500

# Mean: 159441.167

# Std. dev.: 28876.960

# Sample Metadata Categories: None provided

# Observation Metadata Categories: taxonomy

#

#Counts/sample detail:

# 31: 95033.0

# 37: 124903.0

# 30: 130908.0

# 32: 143009.0

# 34: 157279.0

# 33: 165676.0

# 35: 173083.0

# 39: 177722.0

# 40: 178793.0

# 38: 184629.0

# 36: 191000.0

# 41: 191259.0

#==> RF with 95,000

**#Rarefaction (fast, seconds)**

#single step, even sampling d=95,000 seqs/sample:

single_rarefaction.py -i Otu_table_mincount005.biom -o Otu_table_mincount005_RF95000.biom -d 95000

==============================================================================================

**#1. Significance of differences in OTU table (no chimeric seqs and no singletons):**

#Nonparametric t-test:

group_significance.py -i Otu_table.biom -m map.txt -s nonparametric_t_test -c Description -o category_significance_npttest.txt

#Parametric t-test:

group_significance.py -i Otu_table.biom -m map.txt -s parametric_t_test -c Description -o category_significance_ttest.txt

**#2. Summarize data in OTU table, no chimeric seqs and no singletons:**

**#Summarize OTUs based on sampleID but sorted:**

#a) Sort OUT table according to number

sort_otu_table.py -i Otu_table.biom -o Otu_table_sorted_Sort1.biom -m map.txt -s Sort1

#b) Build plots

summarize_taxa_through_plots.py -i Otu_table_sorted_Sort1.biom -o summary_per_sample_sorted_Sort1

#c) Convert from *.biom to *.txt to get read abundances

biom convert -i Otu_table_sorted_Sort1.biom -o Otu_table_sorted_Sort1.biom.txt --to-tsv --header-key taxonomy

**#Summarize OTUs according to Description:**

summarize_taxa_through_plots.py -i Otu_table.biom -m map.txt -s -o summary_Description -c Description

**#Parametric t-test:**

group_significance.py -i summary_per_sample_sorted_Sort1/Otu_table_sorted_Sort1_L2.biom -m map.txt -s parametric_t_test -c Description -o summary_per_sample_sorted_Sort1/category_significance_ttest_L2.txt

group_significance.py -i summary_per_sample_sorted_Sort1/Otu_table_sorted_Sort1_L3.biom -m map.txt -s parametric_t_test -c Description -o summary_per_sample_sorted_Sort1/category_significance_ttest_L3.txt

group_significance.py -i summary_per_sample_sorted_Sort1/Otu_table_sorted_Sort1_L4.biom -m map.txt -s parametric_t_test -c Description -o summary_per_sample_sorted_Sort1/category_significance_ttest_L4.txt

group_significance.py -i summary_per_sample_sorted_Sort1/Otu_table_sorted_Sort1_L5.biom -m map.txt -s parametric_t_test -c Description -o summary_per_sample_sorted_Sort1/category_significance_ttest_L5.txt

group_significance.py -i summary_per_sample_sorted_Sort1/Otu_table_sorted_Sort1_L6.biom -m map.txt -s parametric_t_test -c Description -o summary_per_sample_sorted_Sort1/category_significance_ttest_L6.txt

#Nonparametric t-test:

group_significance.py -i summary_per_sample_sorted_Sort1/Otu_table_sorted_Sort1_L2.biom -m map.txt -s nonparametric_t_test -c Description -o summary_per_sample_sorted_Sort1/category_significance_npttest_L2.txt

group_significance.py -i summary_per_sample_sorted_Sort1/Otu_table_sorted_Sort1_L3.biom -m map.txt -s nonparametric_t_test -c Description -o summary_per_sample_sorted_Sort1/category_significance_npttest_L3.txt

group_significance.py -i summary_per_sample_sorted_Sort1/Otu_table_sorted_Sort1_L4.biom -m map.txt -s nonparametric_t_test -c Description -o summary_per_sample_sorted_Sort1/category_significance_npttest_L4.txt

group_significance.py -i summary_per_sample_sorted_Sort1/Otu_table_sorted_Sort1_L5.biom -m map.txt -s nonparametric_t_test -c Description -o summary_per_sample_sorted_Sort1/category_significance_npttest_L5.txt

group_significance.py -i summary_per_sample_sorted_Sort1/Otu_table_sorted_Sort1_L6.biom -m map.txt -s nonparametric_t_test -c Description -o summary_per_sample_sorted_Sort1/category_significance_npttest_L6.txt

#---------------------------------------------------------------------------------------

**#3. Beta_diversity (Unifrac)**

#This analysis requires:

# a. rarefaction of OTU_table (single rarefaction to have same amount of seqs in each sample)

# b. calculation of b-div

# c. generate principal coordinates

# d. 2D/3D PCoA plots

# e. Distance histograms

# f. UPGMA tree

**#a. Rarefaction (fast, seconds)**

#already performed

**#b. Calculation ß-div (quick, seconds)**

#unweighted Unifrac:

beta_diversity.py -i Otu_table.biom -m unweighted_unifrac -o unweighted_unifrac/ -t tree.tre

#weighted Unifrac:

beta_diversity.py -i Otu_table.biom -m weighted_unifrac -o weighted_unifrac/ -t tree.tre

#Bray-Curtis:

beta_diversity.py -i Otu_table.biom -m bray_curtis -o bray_curtis/ -t tree.tre

**#c. Principal coordinates (quick, seconds)**

#unweighted Unifrac:

principal_coordinates.py -i unweighted_unifrac/unweighted_unifrac_Otu_table.txt -o unweighted_coord.txt

#weighted Unifrac:

principal_coordinates.py -i weighted_unifrac/weighted_unifrac_Otu_table.txt -o weighted_coord.txt

#Bray-Curtis:

principal_coordinates.py -i bray_curtis/bray_curtis_Otu_table.txt -o bray_curtis.txt

**#d. 2D PCoA plots (quick, seconds)**

#2D unweighted:

make_2d_plots.py -i unweighted_coord.txt -m map.txt -b Description -o 2D_plot_unweighted

make_2d_plots.py -i weighted_coord.txt -m map.txt -b Description -o 2D_plot_weighted

make_2d_plots.py -i bray_curtis.txt -m map.txt -b Description -o 2D_plot_bray_curtis

make_emperor.py -i unweighted_coord.txt -m map.txt -o emperor_output_unweighted

make_emperor.py -i weighted_coord.txt -m map.txt -o emperor_output_weighted

make_emperor.py -i bray_curtis.txt -m map.txt -o emperor_output_bray_curtis

compare_categories.py --method permanova -i unweighted_unifrac/unweighted_unifrac_Otu_table.txt -m map.txt -c Description -o permanova_out_unweighted_Description -n 999

compare_categories.py --method adonis -i unweighted_unifrac/unweighted_unifrac_Otu_table.txt -m map.txt -c Description -o adonis_out_unweighted_Description -n 999

compare_categories.py --method anosim -i unweighted_unifrac/unweighted_unifrac_Otu_table.txt -m map.txt -c Description -o anosim_out_unweighted_Description -n 999

compare_categories.py --method mrpp -i unweighted_unifrac/unweighted_unifrac_Otu_table.txt -m map.txt -c Description -o mrpp_out_unweighted_Description -n 999

#---------------------------------------------------------------------------------------

**#4. Alpha_diversity (PD)**

#This analysis requires:

#14a. rarefaction of OTU_table

#14b. calculation of a-div from rarefied tables

#14c. collate results

#14d. make plots

#a. Rarefaction of OTU_table (15 min)

#sample from m=1000 seqs/sample to x=96000 seqs/sample by increasing of s=500 seqs at each step and doing n=3 iterations at each step

multiple_rarefactions.py -i Otu_table.biom -m 1000 -x 101000 -s 5000 -n 3 -o rarefied_a-div_otu_tables/

#b. calculation of a-div from rarefied tables (30 min)

alpha_diversity.py -i rarefied_a-div_otu_tables/ -m PD_whole_tree,chao1,simpson,simpson_e,shannon,observed_otus -o alpha-div/ -t tree.tre

#c. Collate results (quick)

collate_alpha.py -i alpha-div/ -o collated_alpha-div

#d. Make plots (quick)

make_rarefaction_plots.py -i collated_alpha-div -m map.txt -o a-div_plots/
